# Beyond effectiveness evaluation: Contributing to the discussion on complexity of digital health interventions with examples from cancer care

**DOI:** 10.3389/fpubh.2022.883315

**Published:** 2022-07-22

**Authors:** Filipa Ventura, Maria Brovall, Frida Smith

**Affiliations:** ^1^Health Sciences Research Unit: Nursing, Nursing School of Coimbra, Coimbra, Portugal; ^2^Department of Nursing, School of Health and Welfare, Jönköping Academy, Jönköping University, Jönköping, Sweden; ^3^Department of Oncology, Institution of Clinical Sciences, Sahlgrenska Academy, University of Gothenburg, Gothenburg, Sweden; ^4^Regional Cancer Centre West, Western Sweden Healthcare Region, Gothenburg, Sweden; ^5^Department of Technology Management and Economics, Center for Healthcare Improvement, Chalmers University of Technology, Gothenburg, Sweden

**Keywords:** interactive health communication technologies, hybrid designs, complex interventions, cancer care, telehealth

## Abstract

Digital health interventions (DHIs) have become essential complementary solutions in health care to enhance support and communication at a distance, with evidence of improving patient outcomes. Improving clinical outcomes is a major determinant of success in any health intervention, influencing its funding, development, adoption and implementation in real-world practice. In this article we explore our experiences of developing and testing DHIs to identify and discuss complexity challenges along their intervention research lifecycle. Informed by the case study research approach, we selected three individual DHIs aimed at satisfying the supportive and educational needs of people living with cancer. The Care Expert, the Digi-Do and the Gatapp were underpinned on different complexity frameworks i.e., the Medical Research Council framework and the Non-adoption, Abandonment, Scale-up, Spread and Sustainability framework. This variance on the methodological underpinning was expected to prompt a multifaceted discussion on the complexity dimensions endorsed by each of the frameworks. Our discussion endorses the adoption of mixed-methods research designs, to gather the perspectives of stakeholders and end-users, as well as pragmatic evaluation approaches that value effectiveness outcomes as much as process outcomes. Furthermore, the dissemination and sustainability agenda of DHIs needs to be considered from early-stage development with the inclusion of a business model. This business plan should be worked in partnership with healthcare services, regulatory bodies and industry, aiming to assure the management of the DHI throughout time.

## Introduction

Digital health interventions (DHIs) are rapidly gaining clinical importance as essential complementary solutions to enhance support and communication at a distance in healthcare (1). The World Health Organization (2016), defines digital health as the use of digital, mobile and wireless technologies to support the achievement of health outcomes (2).

DHIs are useful across many clinical domains. In cancer care, DHIs allow the provision of self-management support, telemonitoring and health education, in a self-paced process and on-demand. As complementary care resources, DHIs have shown evidence of improving person-relevant outcomes, such as self-efficacy, health competence, and healthcare participation, as well as patient-reported outcomes, such as depression, anxiety, pain, fatigue and wellbeing (3).

It has also been established that very few interventions are truly simple, with scholars arguing for a continuum of simplicity-complexity depending on each complexity dimension (4). A complex health intervention might be defined as dynamic activities containing multiple components, with the potential for interaction among them, and that, when delivered to the intended target audience in a specific context, might lead to a range of possible and variable outcomes (5). Considering the emerging evidence on the processes and mechanisms of action, DHIs will more likely be placed toward the complexity-end of the continuum, posing many challenges, particularly with regards to effectiveness assessment and implementation in the real-world (6).

The potential to improve clinically relevant outcomes is a major determinant of success in any health intervention, influencing possibilities for its funding, development, adoption and implementation in real-world practice. In this article, we explore our experiences of developing and testing DHIs to identify and discuss complexity challenges along their intervention research lifecycle. Informed by the case study research approach, we selected three individual DHIs aimed at satisfying the supportive and educational needs of people living with cancer (7). The Care Expert, the Digi-Do and the Gatapp were conveniently chosen, because they were underpinned by different complexity frameworks yet shared similar intervention goals and target samples. We expected that these similarities would allow our analysis to focus on the complexity dimensions and approaches endorsed by these different frameworks, thus we were guided by the Medical Research Council framework (MRCf) (8) and the Non-adoption, Abandonment, Scale-up, Spread and Sustainability framework (NASSSf) (6).

## Complexity theory applied to the development and evaluation of digital health interventions

The MRCf for developing and evaluating complex interventions in health pioneered the eliciting of complexity elements in health interventions and advocated for rigorous methodological approaches to manage identified uncertainties. The original framework was revised in 2008, depicting a circular process to the intervention research lifecycle, comprising specific stages of development, piloting and feasibility, effectiveness and implementation with feedback loops in between (4). The circularity of the research process was relevant for promoting an equal focus to the different stages of the intervention research, beyond the effectiveness evaluation, which was previously the main focus in the linear version. In 2021, a new update highlighted the relationship between the intervention and its context and emphasized the adoption of diverse research perspectives in intervention research (i.e., efficacy, effectiveness, theory-based and systems). At each stage, the new guidance identifies the importance of accounting for 6 core elements: contextual relevance, adequacy of program theory, engagement of stakeholders, key uncertainties, intervention refinement, and economic adequacy (8).

The NASSSf complements the MRC by considering complexity dimensions specific to health and care technologies as health interventions, particularly concerning their non-adoption, abandonment, scale-up, spread and sustainability. More concretely, the NASSSf supports researchers to predict and evaluate the success of technology-mediated health or social care programs by posing questions within several domains and the interaction and mutual adoption between these domains over time, while highlighting the challenges pertaining to each of the domains. The more complex the domains are considered to be, the harder it is for an intervention to become mainstream in clinical practice (6). As such, the framework aims to support researchers to work with the various stakeholders to identify the key questions about complex interventions, and to design and conduct research with diverse perspectives and an appropriate combination of methods.

## Reflections from intervention research on digital health interventions

Here we present each case in more detail and reflect on the identified complexity elements that, from our perspective, might challenge the traditional effectiveness evaluation designs and the sustainability of DHIs.

Aiming to satisfy specific needs of people living with cancer, we developed three prototypes of DHIs, which we summarize in Table 1. The Care Expert, the Digi-Do and the Gatapp each target women undergoing treatment for breast cancer and have many similarities in the founding theory and delivery medium. The distinguishing feature of each DHI is the complexity framework guiding its development, piloting, effectiveness evaluation and implementation.

**Table 1:** Main features of the individual DHIs projects.

	**Care expert**	**Digi-Do**	**Gatapp**
Target population	Women undergoing treatment for early-stage breast cancer	Women before radiotherapy for breast cancer	Women before radiotherapy for left sided breast cancer
Theory	Person-centered care (9) and Social Support (10)	Person-centered care (9) Health literacy (11)	Person-centered care (9) Health literacy (11)
Complexity framework	MRCf	NASSSf	NASSSf
Delivery medium	Mobile applications	Mobile applications	Mobile application
Intervention lifecycle phase	Usability testing	RCT completed, analysis phase	Pre-studies, prototyping

The Care Expert is an e-supportive system aiming to mediate person-centered care in the context of outpatient oncology. It was developed following the MRCf principles to strengthen women's agency within the care partnership (12). In its current version, The Care Expert app is composed of three individual supportive components, revealing high acceptability in a preliminary usability test (13).

The Digi-Do is a digital information tool to help patients with breast cancer to be involved and prepared before, during and after the start of radiotherapy treatment. It contains a virtual visit to the radiotherapy department using 360 images, maneuvered with Virtual Reality glasses or with a smartphone. Further, it is complemented with information in the form of Q&As, films and weblinks. Its development was conducted in co-design, using participatory design methodology, and the tool is currently in the final stages of evaluation (14, 15). Simultaneously, the development process is being retrospectively assessed using the NASSS-CAT long version.

Departing from our experiences of developing the Digi-Do, we used the NASSSf, complemented with the Quadruple Helix from early-stage design (16). The combination of both frameworks in a participatory design logic was believed to prompt implementation and sustainability in clinical practice. The Gatapp is in the development phase and aims to prepare patients to perform the correct breathing technique during radiotherapy (Deep Inspiration Breath Hold Radiotherapy, DIBH) by using a sensor connected to a mobile application that enables patients to practice at home while awaiting DIBH treatment.

While conducting research at each of the intervention phases, we discovered complexity elements resulting from the interplay between the person, the intervention and the context, regardless of the complexity framework.

The research process leading to The Care Expert was particularly important in eliciting the challenges concerning the effectiveness evaluation of DHIs (17). In the Care Expert project, the time elapsed from the intervention design to the intervention effectiveness evaluation corresponded with the expansion of the Internet as a delivery medium, making the use of CD-ROM obsolete. This concern has been highlighted by other researchers. Specifically, the time for conducting a traditional randomized controlled trial involving technology-mediated interventions is not compatible with the fast pace of today's technological development (18). Moreover, according to the paradata captured from participants' interactions with the application, the patterns of usage were very heterogeneous, leading to uncertainties in determining the optimum intervention dosage.

From our perspective, each person using an intervention will have different motivations, health beliefs, preferences and abilities, which will naturally result in particular ways of participating in the intervention. Accordingly, the exposure to the intervention will vary at the individual level, challenging the establishment of a predefined intervention dosage, upon which the effect of that intervention is determined. Additionally, tailoring mechanisms, aimed at personalized recommendations, are likely to lead to distinct interventions, perhaps with the strength of doses of supportive content varying upon each person's characteristics. Hence, the pathway by which the intervention components contribute to each of the targeted outcomes at the individual level is difficult to pinpoint and therefore to monitor and evaluate.

For intervention delivery medium, the use of the Internet and mobile applications enable great variability in access settings. Because the choice of access settings completely depends on the participant's preferences, heterogeneity will occur naturally. Moreover, accessing the DHI through the Internet might allow participants to be additionally exposed to other sources of knowledge and support than the DHI, which are not active components of the intervention. The accumulated heterogeneity has great potential to influence the intervention delivery per protocol. If adopting a traditional research design, such elements are likely to constitute pitfalls in the effectiveness evaluation of DHIs.

The Digi-Do project involved end-users and stakeholders from the early stages of intervention design. NASSSf was a useful strategy, along with the Quadruple Helix model (16), to engage multi-level stakeholders and map complexity elements across different domains. The empirical work carried out to identify the requirements, needs and preferences of end-users and stakeholders highlighted the ownership and management of the DHI as a complexity element. From our perspective, the ownership and management of DHIs has significant potential to hinder their sustainability beyond the project's lifetime.

This project further led us to reflect on the adequacy of the measurement instruments used to evaluate effectiveness. Our challenge concerned the selection of outcome measures that were sensitive to the intervention and comprehensively captured the multidimensional phenomena (e.g., quality of life, wellbeing). Such endpoints are increasingly being considered equally important to other biometrically oriented outcomes, as they reflect the relevance and adequacy of health interventions to support persons living with a disease (i.e., long-term illness) (19). One possible strategy to capture multidimensional phenomena might entail the use of multiple measurement instruments to assess specific dimensions of the phenomenon. However, such a strategy might raise the response burden to the extent where adherence to the intervention, attrition and person-relevance might be at risk, consequently jeopardizing the effectiveness evaluation.

We also believe that the adequacy of matching the measurement instruments' content to the phenomena in current society might be a concern. The measurement instruments regularly used to evaluate effectiveness in clinical trials have been developed and tested for many years. Although revealing good reliability and fit, the conceptual equivalence of the phenomenon of attention in current society should be considered and explored alongside each measurement. Quality of life might be an example of such a phenomenon, with different interpretations over time and cultures, particularly considering advancements in medicine and technology.

## Working sustainability from early-stage development

From our reflections on these challenges, along with the research on the selected DIHs, we elicited several cornerstones that we believe are crucial to a DHIs' sustainability beyond a project's lifetime.

### Engaging end-users and stakeholders

Involving end-users and stakeholders throughout the different stages of research across clinical domains is recognized as a gold-standard and an ethical duty (20). Taking oncology as an example, the European Council has developed principles of successful patient involvement in cancer research (21). These acknowledge the importance of strong patient involvement and accountability for patient experience throughout cancer research processes. Five further considerations for the successful involvement of people living with cancer in research are also recommended: (i) strategy, level and timing, (ii) communication, understanding and relationships, (iii) resources, knowledge and skills, (iv) methods and approaches, and (v) ethical and legal aspects (21).

Involving patients in research goes beyond merely informing the research process on the real needs. It tackles questions concerning when and how the research on those needs should be undertaken. In complex health intervention research, involving patients in research has led to increased acceptability and adherence to interventions among patients and healthcare professionals (8). For DHIs, having end-users and stakeholders as research partners from early design to late implementation follow-up is one of the cornerstones of developing usable, used and useful interventions, adding value to standard care (8).

The vast number of strategies found in the methodological literature to enable such involvement reflects efforts to promote the communication, training and standardization of methods for involvement. Among them, participatory design (PD) has received particular attention in health technology research (22). PD approaches enable the engagement of end-users and stakeholders in creative and reflexive processes throughout the intervention research cycle, ultimately resulting in an artifact (e.g., website, MedTech device or app) (23). PD approaches might assist health researchers to plan and implement activities that promote the exploration of the specificities of DHIs in a rigorous and systematic way from the perspectives of all involved (24).

### Establishing ownership

From our perspective, working on a business plan from an early stage is crucial for enhancing sustainability and spread of DHIs. A consensual strategy must be developed among the many stakeholders for the successful utilization, management and maintenance of the DHI beyond the project's lifetime and in clinical practice. Here, the Quadruple Helix model explored in a participatory logic might be an asset.

When considering innovation through health technology in current society, the relationship between academia, industry, regulatory bodies and civil society must be considered (25). The Quadruple Helix model highlights the social responsibility of innovation, thereby reinforcing the involvement of citizens in the research and development of technology. Evidence shows that the Quadruple Helix is an adequate model to explore innovation development with end-users and stakeholders and its sustainable translation into society (26), making it suitable for including the perspectives of developers (i.e., industry, healthcare services, and research centers), end-users (i.e., patients and healthcare professionals) and authorities (i.e., regulatory bodies, healthcare services administrators) (16).

### Accounting for the context

From our perspective, equally important to ascertain whether an intervention works is to understand when it works, for whom and how, particularly in relation to the existing treatment and care journeys. Mastering the available evidence, the intervention theory and the context within which the intervention unfolds, is crucial for anticipating real-world contingencies in an effectiveness evaluation (6).

Moreover, conducting process evaluation alongside effectiveness trials is essential to understand the outcomes in light of the pathways leading to them (27). Such a strategy will likely inform the implementation process to more accurately fit the clinical context in which the DHI will unfold and highlight the complexity elements arising from the interplay between the DHI and the context.

Given that the specificities of self-paced access to DHI is dependent upon the participants' preference or need (e.g., patient's home, work, free-time setting), the traditional concept of context might need a reformulation. From our perspective, the context should comprise the setting in which the DHI is accessed, as well as the multi-level context where the treatment and care journey occur (e.g., outpatient care pathway). Accordingly, the multidisciplinary healthcare team, the organizational processes and the wider healthcare service are elements that must be carefully considered throughout the intervention research process. Here, the NASSSf offers systematic guidance to support the identification of complexity elements across micro-, meso- and macro domains of health technology innovation (6).

## Future research and practice

The evidence on complex health interventions and DHIs reinforces the need to adopt research designs that enable exploring phenomena alongside the person experiencing them, and in their daily living contexts. Such an understanding might be enhanced through qualitative methods as a complement to quantitative approaches to evaluation, regardless of their focus on effectiveness or processes.

From our experiences, ensuring participation of all involved throughout the research process is crucial. Such involvement comprises the interpreting and acting phases and should occur in an academy-community collaborative processes that are endorsed by action research principles, designed to strengthen the richness, rigor and relevance of interventions for their users in their context (28).

Mixed-methods approaches are likely to allow us to elicit unique needs during DHI design and development by describing the effects with numbers and forming an understanding of the success or failure of implementation efforts (29). Aligned with this, the Most Significant Change Technique (30) has received attention in recent years as an innovative tool for monitoring and evaluating complex health interventions (31). The collection and discussion of stakeholders' stories of significant change regularly throughout the project allows for adaptative management (31). This technique might further support the establishment of the ownership of DHIs and is promising as a complementary effectiveness evaluation approach.

Given the many elements that DHIs portray, variability and heterogeneity are inevitable, and this might reduce their external validity. From our perspective, we need methodological strategies that enhance DHIs' sustainability and spread without reducing their internal validity. These principles position us in the realm of pragmatic trials (32). A pragmatic trial design allows for effectiveness evaluation methods that consider personal and contextual elements with the main goal of enhancing the knowledge transfer between settings. Pragmatic trials are likely to more adequately inform an adaptation or scale-up of the intervention to new contexts. In this sense, we move toward the realistic paradigm and its principles.

Ultimately, the alliance of paradigms should be considered throughout the intervention research cycle. Thoughtful reflection should consider their suitability for tackling the identified complexity elements, while rigorously and comprehensively addressing the phenomenon from the perspective of the people experiencing them and in context.

The transferability of our reflections and discussion should consider the sample of cases selected to inform this article. We based our analysis on three cases that had more commonalities than differences. Considering more cases with more differences would likely have led us to more in-depth and consolidated reflections. Moreover, as each is situated in the oncology domain, a judgment on the transferability of the complexity issues to other clinical areas cannot be made. Although further cases would be needed to inform that judgment, we believe that the complexity issues identified here are not specific to cancer care, they are rather related to the interplay between DHIs and the real-world context more broadly. The most significant difference between the selected DHIs, i.e., the complexity framework, is, from our perspective, a strength of the analysis as it allowed the discussion to focus on multifaceted complexity elements.

## Conclusion

This analysis of the identified challenges endorses the adoption of mixed-methods research designs to gather the perspectives of stakeholders and end-users, as well as pragmatic evaluation approaches that value effectiveness outcomes as much as process outcomes. Furthermore, the dissemination and sustainability agenda of DHIs must be considered from early-stage development with the inclusion of a business model. This business plan should be worked in partnership with healthcare services, regulatory bodies and industry, aiming to assure the management of the DHI over time.

DHIs are helpful and effectively complement healthcare. Yet high-quality research is still demanded. Methodological rigor must be maintained throughout the research lifecycle. Strategies to improve patient and health professional engagement in the design and delivery of these interventions must be put in place. DHIs entail many complexity dimensions that demand cooperative efforts and varied expertise. Such combinations should go beyond disciplinary boundaries to enable the successful design, evaluation, implementation and sustainability of DHIs.

## Data availability statement

The original contributions presented in the study are included in the article, further inquiries can be directed to the corresponding author/s.

## Author contributions

FV led the writing of the perspective article. All authors contributed equally to the idea conception, design, and discussion. All authors contributed with important intellectual content to the perspective article and approved the final version for publication.

## Funding

This study is partially funded by National Funds through the FCT – Foundation for Science and Technology, I.P., within the scope of the project Refª. UIDB/00742/2020. The study of FV was funded by FCT, CEECINST/00103/2018. The funders had no role in the article.

## Conflict of interest

The authors declare that the research was conducted in the absence of any commercial or financial relationships that could be construed as a potential conflict of interest.

## Publisher's note

All claims expressed in this article are solely those of the authors and do not necessarily represent those of their affiliated organizations, or those of the publisher, the editors and the reviewers. Any product that may be evaluated in this article, or claim that may be made by its manufacturer, is not guaranteed or endorsed by the publisher.
