# Corrigendum: Beyond effectiveness evaluation: Contributing to the discussion on complexity of digital health interventions with examples from cancer care

**DOI:** 10.3389/fpubh.2022.1049512

**Published:** 2022-10-19

**Authors:** Filipa Ventura, Maria Brovall, Frida Smith

**Affiliations:** ^1^Health Sciences Research Unit: Nursing, Nursing School of Coimbra, Coimbra, Portugal; ^2^Department of Nursing, School of Health and Welfare, Jönköping Academy, Jönköping University, Jönköping, Sweden; ^3^Department of Oncology, Institution of Clinical Sciences, Sahlgrenska Academy, University of Gothenburg, Gothenburg, Sweden; ^4^Regional Cancer Centre West, Western Sweden Healthcare Region, Gothenburg, Sweden; ^5^Department of Technology Management and Economics, Center for Healthcare Improvement, Chalmers University of Technology, Gothenburg, Sweden

**Keywords:** interactive health communication technologies, hybrid designs, complex interventions, cancer care, telehealth

In the published article, there was an error in the Funding statement. The funding statement displayed the Health Sciences Research Unit- Nursing (UICISA: E) hosted by the Nursing School of Coimbra (ESEnfC), Coimbra, Portugal as one of the funding parties, as follows “This article processing charges and submission fees were partially commissioned by the Health Sciences Research Unit- Nursing (UICISA: E) hosted by the Nursing School of Coimbra (ESEnfC), Coimbra, Portugal.” The correct Funding statement appears below.

## Funding

This study is partially funded by National Funds through the FCT - Foundation for Science and Technology, I.P., within the scope of the project Refª. UIDB/00742/2020. The study of FV was funded by FCT, CEECINST/00103/2018. The funders had no role in the article.

The authors apologize for this error and state that this does not change the scientific conclusions of the article in any way. The original article has been updated.

## Publisher's note

All claims expressed in this article are solely those of the authors and do not necessarily represent those of their affiliated organizations, or those of the publisher, the editors and the reviewers. Any product that may be evaluated in this article, or claim that may be made by its manufacturer, is not guaranteed or endorsed by the publisher.

